# Knotted vs. Unknotted Proteins: Evidence of Knot-Promoting Loops

**DOI:** 10.1371/journal.pcbi.1000864

**Published:** 2010-07-29

**Authors:** Raffaello Potestio, Cristian Micheletti, Henri Orland

**Affiliations:** 1 SISSA - Scuola Internazionale Superiore di Studi Avanzati, Trieste, Italy; 2 DEMOCRITOS CNR-IOM, Trieste, Italy; 3 Italian Institute of Technology (SISSA unit), Trieste, Italy; 4 Institut de Physique Théorique, CEA, Gif-sur-Yvette, France; National Cancer Institute, United States of America and Tel Aviv University, Israel

## Abstract

Knotted proteins, because of their ability to fold reversibly in the same topologically entangled conformation, are the object of an increasing number of experimental and theoretical studies. The aim of the present investigation is to assess, on the basis of presently available structural data, the extent to which knotted proteins are isolated instances in sequence or structure space, and to use comparative schemes to understand whether specific protein segments can be associated to the occurrence of a knot in the native state. A significant sequence homology is found among a sizeable group of knotted and unknotted proteins. In this family, knotted members occupy a primary sub-branch of the phylogenetic tree and differ from unknotted ones only by additional loop segments. These “knot-promoting” loops, whose virtual bridging eliminates the knot, are found in various types of knotted proteins. Valuable insight into how knots form, or are encoded, in proteins could be obtained by targeting these regions in future computational studies or excision experiments.

## Author Summary

Out of the tens of thousands of known protein structures, only a few hundred are knotted. The latter epitomize, better than unknotted proteins, the degree of coordinated motion of the backbone required to fold reversibly in a specific native conformation, which indeed must contain a precise knot in a specific protein region. In the present work we search for salient features associated to protein “knottedness” through a systematic sequence and structure comparison of knotted and unknotted protein chains. A significant sequence relatedness is found within a sizeable group of knotted and unknotted proteins. Their tree of sequence relatedness suggests that the knotted entries all diverged from a specific evolutionary event. The systematic structural comparison further indicates that the knottedness of several different types of proteins is likely ascribable to the presence of short “knot-promoting” loops. These segments, whose bridging eliminates the knot, are natural candidates for future experimental/computational studies aimed at clarifying whether the global knotted state of a protein is influenced by specific regions of the primary sequence.

## Introduction

Since the early 90's, when the first crystal structures of knotted proteins became available, the number of known knotted protein chains has increased to comprise several hundred PDB [1] instances spanning a few different folds and functional families [2], .

Even before the discovery of knotted proteins, the possible existence of non-trivial topological entanglements, or lack thereof, in proteins was a matter of debate [4], [5]. From a general polymer physics point of view, sufficiently long heteropolymers in canonical equilibrium would be expected to be highly knotted [6]–[10]. The quantitative theoretical estimates of the fraction of knotted molecules hold well for biopolymers such as DNA in a variety of physical situations [11]–[15]. Yet, these estimates cannot be extended to naturally-occurring proteins where the incidence of knots is far lower than what expected for randomly-collapsed flexible polymers [16]. The discrepancy, may reflect the action of several evolutionary mechanisms that have arguably accompanied the selection of viable protein folds.

In support of this view it should be stressed that proteins differ from globular flexible polymers not only in terms of the low incidence of knots but especially because, in the absence of any specific cellular machinery, the same knot type is formed reversibly and reproducibly in the same protein location [17], [18]. This experimental fact poses several conceptual challenges particularly regarding the relationship between the interplay of local folding events and the highly non-local degree of coordination that is intuitively required to tie a given knot in a certain protein position.

These considerations have stimulated an increasing number of experimental and theoretical studies aimed at understanding the kinetic and thermodynamic processes leading to knot formation in proteins or the implications for the molecular mechanical stability [2], [3], [17]–[27]. Specifically, numerical studies employing steered molecular dynamics towards the native state have shown that knotted structures are less accessible targets compared to generic unknotted ones [3], [26]. Furthermore, it was suggested that knots are formed from a single (and local) loop threading event [3], [26]. At the same time experiments [17], [18] indicate that the knot formation process is not hindered by the presence, at the protein's termini, of large, structured, additional chains. This suggests the existence of a global coordination of the protein chain dynamics while still unfolded [2], [18], [20], [21], [23]. In line with this view, computational studies [22] indicate non-native interactions between highly-hydrophobic segments as possible driving forces enhancing the dynamical accessibility of a knotted native state.

The present work aims at complementing the insight offered by these studies through a systematic quantitative comparative investigation of knotted and unknotted proteins.

Our first aim is to assess, on the basis of available PDB entries, the level of sequence and structure discontinuity between knotted and unknotted proteins. The question is tackled by means of a systematic search of significant sequence- and structure-based correspondences between knotted and unknotted protein pairs. The second aim is to obtain clues about the possible mechanisms leading to the formation of knotted native states by searching for salient systematic differences between knotted/unknotted protein pairs.

Indeed, the PDB-wide sequence and structural comparison indicates that various types of protein knots are associated to the presence of loop segments that are absent from sequence-homologous or structurally-similar unknotted proteins. The removal (virtual bridging) of these segments, which include a region of a knotted transcarbamylase previously identified by Virnau *et al.* [19], manifestly results in unknotted configurations, thus suggesting that the protein segments corresponding to these “knot-promoting” regions have a direct impact on the protein knotted state.

Based on these observation it can be expected that valuable insight into the way that knots form, or are encoded, in proteins could be obtained by targeting these regions in future *in vitro* experiments or with numerical computations.

## Results/Discussion

### Identification of knotted proteins representatives

The 1.2 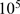 protein chains contained in PDB entries as of December 2009 were processed to establish their knotted or unknotted state. Out of this initially-large number of chains only 247 (from 229 distinct PDB entries) were identified as being knotted. The list, provided in Table S1, has broad overlaps with previously-published tables of knotted proteins [19], [28] based on knot detection criteria different from the one adopted here, see Materials and Methods section.

The set of all knotted proteins found in the PDB is highly redundant; for example, as many as 194 of the 229 knotted proteins, are carbonic anhydrases. The primary sequence comparison of the entries revealed that less than 50 chains are non-identical in sequence. The dataset was hence processed to achieve a uniform, minimally-redundant, coverage in sequence space. The culling procedure returned 11 representative knotted chains, which are listed in Table 1 along with their salient structural and functional characteristics.

**Table 1:** Knotted proteins representatives list.

Name	PDB	Knot type	CATH	EC	Knotted Region
hypothetical protein	2efvA	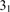 l			6–86
plasmid pTiC58 VirC2	2rh3A	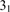 l			82–194
N-succinyl-L-ornithine transcarbamylase (SOTCase)	2fg6C	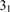 r	01:3.40.50.1370 **02:3.40.50.1370**		149–257
methyltransferase (MT) domain of human TAR (HIV-1) RNA binding protein (TARBP1)	2ha8A	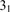 r			83–167
alpha subunit of human S-adenosyl-methionine synthetase (SAM-S)	2p02A	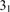 r	01:3.30.300.10 02:3.30.300.10 03:3.30.300.10	2.5.1.6	38–328
human carbonic anhydrase II (CA2)	5cacA	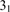 r	3.10.200.10	4.2.1.1	11–260
acetohydroxyacid isomeroreductase	1qmgA	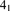	01:3.40.50.720 **02:1.10.1040.10**	1.1.1.86	302–553
photosensory core domain of aeruginosa bacteriophytochrome (PaBphP)	3c2wH	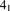			5–302
ubiquitin carboxy-terminal hydrolase (UCH)	2etlA	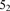 l	3.40.532.10	3.4.19.12	1–233
group I haloacid dehalogenase	3bjxB	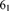 r		3.8.1.10	46–288
ribosomal 80S-eEF2-sordarin complex	1s1hI	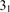 r			78–125

List of the knotted protein representatives. CATH [39] and EC [57] codes are indicated where available; the knotted region refers to the PDB residue numbering. The chirality is indicated with a 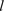 or 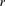 tag appended to the knot type. CATH domains containing the knot are highlighted in boldface for multidomain proteins. The knot region is defined by taking the strictly knotted protein segment returned by the Protein Knot server [50] and extending it by 20 amino acids on both sides. For protein chain 2p02A, which is not recognised as knotted by the server, the strictly knotted protein segment was identified using the method of ref. [58]. The knot in the last entry (1s1hI) has a probably artefactual origin, see Results and Discussion.

### Knots spectrum and knot chirality

The simplest knot type, 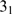, also known as trefoil knot, is by far the most abundant knot type in the initial redundant set, and is also the most abundant in the representative list of Table 1. Indeed, 7 of the 11 entries are trefoils.

Among the trefoil representatives in Table 1 we have identified the shortest known knot, consisting of only 10 amino acids. The knot is found in the cryo-em resolved PDB entry 1s1hI (ribosomal 80S-eEF2-sordarin complex) [29]. Several clues point to its possible artifactual nature: the knotted region (from a.a. 98 to 105) is listed in the structure file as having highly non-standard stereochemical parameters. Furthermore, the associated temperature-factor values are in excess of 100, and are hence indicative of poor compliance with the electron-density map. For these reasons the knot in entry 1s1hI is probably artifactual and will be excluded from further considerations.

The more complex knot types, 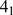, 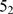, are represented by two and one entries respectively in Table 1 and, in any case, by very few chains in the redundant set. The survey of the December 2009 PDB release did not return knots more complex than the 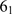 type, which was recently reported in ref. [3].

It is interesting to observe a parallel between the chronological succession of the first PDB release of the various types of protein knots and the complexity of the knots. In fact, the first structures containing 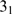, 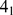, 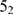 and 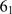 knots were resolved or released, respectively, in 1988 (PDB entries 4cac and 5cac [30]), 1996 (PDB entry 1yve [31]), 2004 (PDB entry 1xd3 [32]) and 2007 (PDB entry 3bjx [33]). Although the steady increase of the PDB cannot be viewed as resulting from the repeated addition of structures sampled uniformly in “protein structure space”, it is natural to assume that the chronological succession of the knots “discovery” is inversely correlated to the abundance of the various knot types.

This qualitative consideration is supported by the fact that, in compact flexible polymers, the abundance of the simplest knot types decreases with knot complexity [12], [34]. One notable point of these polymeric reference systems is that, for entropic reasons, the knot type 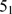 is appreciably less abundant than 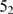, which has the same nominal complexity [12], [13]. The absence of the 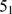 knot in presently-available proteins (a fact previously also related to the unknotting number [2]), may thus reflect the still limited pool of known knotted proteins and might hence populate in the future.

Finally, we discuss the extent to which knots of different handedness occur among knotted proteins. Apart from the 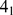 knot which is achiral, knots 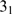, 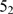 and 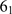 can exist in left- and right-handed versions. Previous observations made on a redundant set of proteins folded in trefoil knots concluded that, except for a single protein entry, all other ones were right-handed trefoils. For the most numerous family of knotted proteins, namely carbonic anhydrases, the bias towards right-handed knots was related to the intrinsic chirality of the 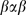 motif adopted by such enzymes [2].

The investigation of the handedness in this latest dataset, where sequence redundancy has been removed, provides a novel context for examining the problem. As reported in Table 1, the balance between right- and left-handed knots is 5 to 3, respectively. The near equality of the populations is thus compatible with the null hypothesis that left- and right-handed protein knots occur in equal proportion (after removal of the biases of representation due to sequence redundancy of otherwise detectable evolutionary relationships).

### Sequence→structure relationship

Simulations of the protein folding of knotted proteins, based on simplified steered dynamics targeted towards the known native state, have reported a much lower degree of efficiency in reaching the native state from an extended conformation compared to unknotted proteins [3], [22], [26]. This difference could be associated to the expectedly higher level of protein motion coordination required to fold correctly in a knotted conformation versus an unknotted one. One would therefore conclude that the topological property of being knotted takes the difficulty of the folding process to a level that is considerably more challenging than for unknotted proteins.

This consideration is here taken as the motivation for a systematic survey of whether, and to what extent, knotted proteins are discontinuously related by sequence and structure to unknotted ones.

In this section we tackle one facet of the problem. Specifically, we shall examine how primary-sequence similarities reverberate in relatedness of the knotted/unknotted topological state. To this purpose, for each of the 11 representatives in Table 1 we performed a PDB-wide BLAST [35] search for related sequences. The search was restricted to sequences of proteins of known structure (i.e. contained in the PDB) because without the structural data it would not be possible to compare the knottedness of pairs with related primary sequences.

The BLAST queries were run with a stringent E-value threshold (0.1) for returned matches, so that false positives are not expected to occur appreciably among the returned entries. Only for three protein chains, namely 5cacA, 2fg6C and 2ha8A, the number of significant matches was larger or equal to 10. Incidentally we mention that, consistently with the probable artifactual origin of the knot in entry 1s1hI, all the 10 significant BLAST matches of 1s1hI were unknotted protein chains.

All the returned matches for the 5cacA human carbonic anhydrase and the 2ha8A methyltransferase domain of the human TAR RNA binding protein (TARBP1-MTd), consisted esclusively of a dozen knotted proteins, all with the same knot type. These matches are therefore not informative for the purpose of understanding if and how differences in sequence reverberate into differences of knotted state.

On the contrary, the BLAST matches of the trefoil-knotted N-succinyl-ornithine transcarbamylase (SOTCase), associated to the PDB entry 2fg6C [36], proved particularly interesting as only 7 of the tens of matching entries are knotted (all in a trefoil knot).

To advance the understanding of the precise type of sequence relatedness of the SOTCase and its knotted and unknotted homologs, the matching BLAST sequences were used as input for a CLUSTALW multiple sequence alignment [37]. The results were used, in turn, to establish a phylogenetic relationship between the related proteins using a neighbour-joining bootstrapping algorithm [38]. The method associates to each branch of the phylogenetic tree a percent confidence estimated from the occurrence of the branch in 1000 repeated phylogenetic reconstructions using only a subset of the aligned amino acids.

**Figure 1:**
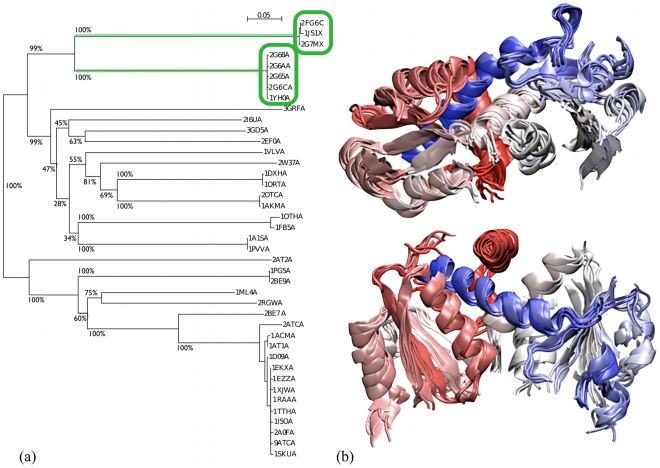
SOTCase and homologous proteins: phylogenetic tree and structural alignment core. (a) The phylogenetic tree was obtained by applying a neighbor joining algorithm [38] to the CLUSTALW multiple sequence alignment of SOTCase and its sequence homologs. The branches' length reflects the percentage sequence dissimilarity (5% gauge shown at the top). The numbers at the nodes, calculated by the bootstrap algorithm, indicate the percent robustness of the separation of two bifurcating branches. The two branches involving knotted proteins (all trefoils) are highlighted in green. (b) Two orthogonal views of the MISTRAL alignment core of six representatives of the SOTCase homologous proteins, namely 2fg6C (knotted), 2i6uA, 2g68A, 2at2A, 1pg5A and 1ortA. These proteins are 313 amino acids long on average. Their alignment core consists of 212 amino acids at an average RMSD of 1.9Å. The color scheme red
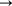
white
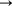
blue follows the N to C sequence directionality. The rendering of PDB structures was carried out using the VMD [56] software.

The phylogenetic tree for the SOTCase is represented in Fig. 1a. The tree shows that the knotted entries appear in two terminal branches sharing a common root. Each branch gathers entries that are highly similar in sequence; in fact their sequence identity (computed by dividing the number of aligned identical amino acids by the average length of the two compared proteins) is not smaller than 90%. The sequence identity across the two branches has the much smaller, but still significant, average value of 40%. The homology relation among all members of the phylogenetic tree is further confirmed by the fact that those, for which CATH [39] code is known, belong to the same CATH family. On the other hand, the robustness of the separation of the knotted sequence subgroup from the unknotted one is strongly suggested by the bootstrap algorithm, with a confidence level larger than 99%.

Amongst the knotted and unknotted entries, the average level of sequence identity is about 20%, with a standard deviation of 7%. Indeed, it is interesting to observe that few knotted/unknotted pairs can have a level of mutual sequence identity even larger than knotted pairs. For example the knotted chain 2g68A has a sequence identity of 33% and 38% respectively, against 1js1X (knotted) and 1pvvA (unknotted).

As, to the best of our knowledge, no previous study had pointed out meaningful relationships of knotted and unknotted proteins, the present results offer a novel insight into the possible mechanisms that have led to the appearance of knotted proteins.

In particular, the phylogenetic tree structure suggests the existence of a simple evolutionary lineage between the sets of knotted and unknotted proteins shown in Fig. 1a. In fact, both groups of trefoil knotted proteins, which have a limited mutual sequence identity, appear to have commonly diverged from the main tree of unknotted entries. The implications are twofold. On the one hand, the robust conservation of the knotted fold in the two sequence-diverged knotted groups suggest the functionally-oriented characteristics of the knotted topology. Indeed, it had already been pointed out for one member of this family, see ref. [19], that the active site is located close to the knotted region, a fact that led to speculate that knottedness would confer a necessary mechanical rigidity to the protein as a whole or to the active site [24], [25]. On the other hand, the existence of a single knotted branch indicates that the knot appearance, and its subsequent conservation, are rare evolutionary events.

Further clues about the biological rationale behind the evolutionary pathways that have led to the emergence/conservation of the knotted structures in Fig. 1a ought to be addressed using more powerful tools than the present sequence-based analysis, in particular, a more general reconstruction of the phylogenetic relatedness should be accomplished within a genome-wide perspective for the organisms involved.

### “Knot-promoting” loops in SOTCase

Valuable insight into the fundamental similarities and differences in the entries appearing in the tree of Fig. 1a can be obtained by inspecting their structural alignment.

To this purpose we used the MISTRAL [40] multiple structural alignment web server which was recently developed by some of us. The use of this multiple structural alignment method, which is non-sequential, appears to be particularly appropriate, since correspondences are sought between proteins with different knotted state, and hence with expected differences in fold organization.

The proteins appearing in the phylogenetic tree can be all simultaneously structurally-aligned. Their aligned core consists of as many as 192 amino acids, which is a substantial fraction of the full proteins (which have an average length of about 310 a.a.). Over the core region, the average RMSD of any pair of matching amino acids is less than 2 Å. The good structural superposability of the protein set (which we recall includes protein pairs with average mutual sequence identity of about 20%) is exemplified in Fig. 1b where the alignment of 6 proteins taken from the various primary branches of the phylogenetic tree is shown.

The detailed pairwise structural comparison indicates that members of the two knotted branches admit a good structural superposition over the full protein length (and, in particular, over the knotted region).

To highlight the salient differences between the knotted and unknotted entries in the tree we analysed all the pairwise structural superpositions of the knotted SOTCase with the unknotted homologs. This investigation generalises the structural comparative inspection of two specific instances of knotted and unknotted carbamylases carried out in ref. [19].

The results are best illustrated considering the closest matching pair, namely the SOTCase and PDB entry 1ortA.

**Figure 2:**
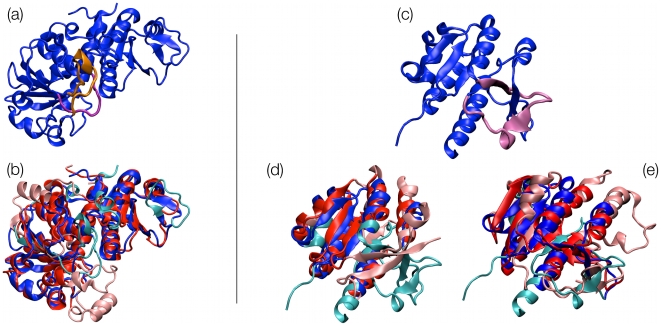
Structural alignment of knotted and unknotted proteins. SOTCase (a) is shown in cartoon representation; the knot-promoting loop segments are highlighted in orange and purple. The MISTRAL alignment with unknotted entry 1ortA is shown in panel (b): aligned residues are colored in blue and red, respectively, while non aligned residues are correspondingly colored in cyan and pink. Knotted protein TARBP1-MTd is shown in panel (c) with the knot-promoting loop segment highlighted in purple. The MISTRAL alignments of TARBP1-MTd with the unknotted proteins 1b93A and 1hdoA are shown in panels (d) and (e), respectively.

In spite of their limited mutual sequence identity, which is about 25%, these proteins admit a very good structural superposition, see Fig. 2a,b. Indeed, as many as 246 of their amino acids (which are 321 and 335 in total for chains SOTCase and chain 1ortA, respectively) can be superposed with an RMSD as small as 2.5Å. The alignment respects the overall sequence directionality of the chains. The few non-matching regions are typically insertions in exposed stretches of the sequence, corresponding to small loops protruding out of the surface of the molecule, which have no particular bearing on the protein topology.

The case is different for two regions of the SOTCase: the proline-rich segment comprising amino acids 174–182, and the segment 235–255; both regions are located in proximity of the active site (residues 176–178, 252). As shown in Fig. 2a, these loops, which do not contain highly hydrophobic segments (see Figure S1), have a particular mutual concatenation which directly impacts on the protein knotted state. In fact, the virtual excision (bridging) of these two segments, which both have a small end-to-end separation, results in the elimination of the knot from SOTCase.

We remark that Virnau *et al.* [19] had recently observed that the knottedness of the transcarbamylase of *X. Campestris* was probably due to the excess length of the region comprising a.a. 176 with respect to the human analog. This observation is reinforced by the present general sequence- and structure-based systematic comparison which additionally points out the systematic absence of a second loop segment 235–255 in the unknotted homologs of the SOTCase. The results provide a quantitative basis for suggesting that some light on the process of protein knot formation can be shed by targeting these regions in suitable mutagenesis experiments. It would be particularly interesting to analyse whether both of the identified “knot-promoting” loops need to be excised to produce an unknotted native state, or if only one would suffice.

### Knot-promoting loops in other proteins

The results discussed in the previous section indicate that knotted proteins appear to be sparsely distributed in sequence space. In fact, only for one of the representatives in Table 1, it was possible to establish significant sequence-based relationships with unknotted proteins.

Here we investigate whether, irrespective of the level of primary sequence relatedness, there exist meaningful structural similarities between knotted and unknotted proteins.

The search was performed, by carrying out MISTRAL structural alignments of each of the knotted representatives in Table 1, against an extensive set of about 2.4 10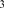 unknotted protein chains. The latter set was obtained by culling the full set of all available PDB chains as of December 2009 using standard criteria based on mutual sequence identity, see Materials and Methods section. The top-ranking alignments are reported in Table S2.

Hereafter we focus on a limited number of cases which, regardless of their ranking in alignment quality, can be aptly used to highlight interesting relationships between knotted and unknotted pairs. In particular, they might possibly be used to shed light on important kinetic or thermodynamic mechanisms that guide or otherwise favor the formation of knots in naturally occurring proteins.

In particular, we start by discussing the limited number of cases where the alignment suggests the presence of knot-promoting loop segments, analogously to the case of the SOTCase and chain 1ortA. These segments are identified using two main criteria: (i) the segments ends must be sufficiently close that they could be virtually bridged by very few amino acids; (ii) the bridging/excision operation should lead to an unknotted conformation.

The automated search for such segments returned positive matches for three representatives. One of them was the same SOTCase chain, which we discussed in previous sections. The other chains were the aforementioned TARBP1-MTd and the photosensory core module of *Pseudomonas aeruginosa* bacteriophytochrome (PaBphP, PDBid 3c2wH).

### TARBP1 methyltransferase domain

TARBP1-MTd aligns well with two unknotted protein representatives that have very different overall structural organization. Despite the differences, discussed hereafter, the alignments consistently indicate that loop 101–123 is a knot-promoting loop for chain A of TARBP1-MTd.

The alignment against the unknotted protein chain 1b93A [41] comprises 87 amino acids (at 3.5 Å RMSD) and covers the entire knotted region with the exception of the above mentioned segment. The fact that the ends of the segments are less than 5Å apart, readily suggests that the excision of the fragment ought to result in an unknotted protein with structure analogous to the 1b93A chain. The inspection of the hydrophobicity profile based on the Kyte and Doolittle scale [42] (see Figure S2) indicates that one of the regions with high hydrophobicity falls within the knot-promoting loop. In analogy with what suggested in ref. [22] for YibK, it is therefore possible that the kinetic accessibility of the knotted state is enhanced by contacts that this region forms with other parts of the protein.

**Figure 3:**
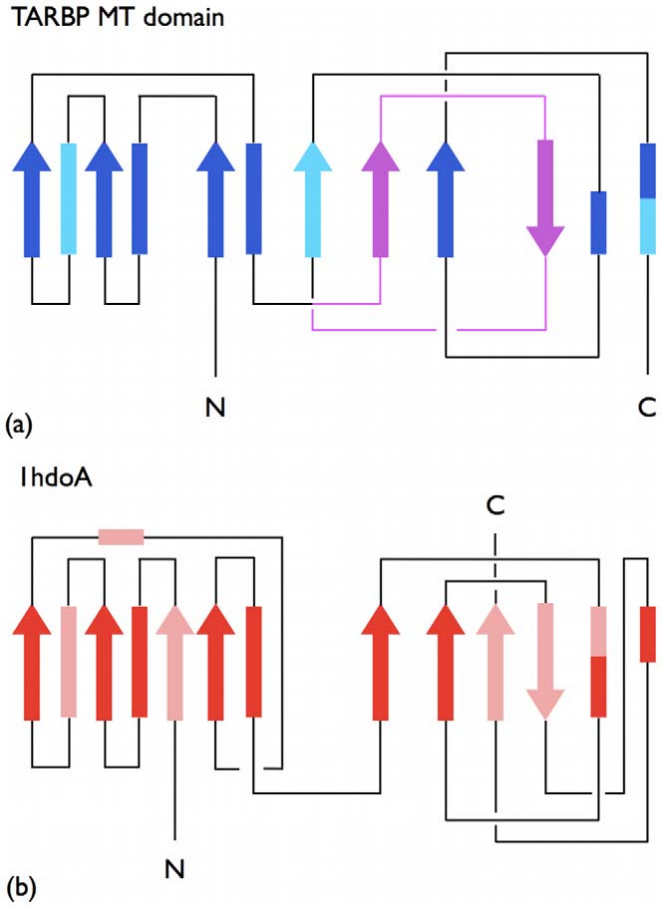
Two-dimensional schematic diagrams. The secondary and tertiary organization of the knotted TARBP1-MTd (PDBid 2ha8A) (a) and unknotted protein chain 1hdoA (b), which admit a significant structural superposability, see Fig. 2. The color-coding of the aligned and non-aligned secondary elements and of the knot-promoting loop follows the one in Fig. 2. The overall correspondence of the secondary elements is manifest, despite noticeable differences in their “wiring” which reflect in (i) a different fold organization and (ii) different knotted state.

The topologically-important role of the segment is further highlighted by the alignment with the 1hdoA chain. At variance with the case of 1b93A, the good alignment does not involve regions that have the same succession, along the primary sequence, in the two proteins. This is readily ascertained by the inspection of the structural diagram of Fig. 3a,b where it is possible to appreciate the different “rewiring” of several corresponding secondary structure elements. In this case too, the alignment comprises the knotted region with the exception of the previously mentioned segment. This reinforces the previous suggestion that the removal of the segment ought to result in an unknotted folded configuration.

### PaBphP photosensory core module

The “figure-of-eight” knot in protein PaBphP [43] spans a very large portion of the photosensory core module of PaBphP (a.a. 24 to 282). This protein is composed of three domains: named PAS (Per-ARNT-Sim), GAF (cGMP phosphodiesterase/adenyl cyclase/FhlA) and PHY (phytochrome) domains. The GAF domain is known to be present in several sequence-unrelated proteins and, in fact, it represent the core region of the good alignment of PaBphP photosensory core module with the non-homologous chain 2b18A [44].

The alignment singles out the segment of amino acids 203 to 256 as a knot-promoting loop. Indeed, while the knot length is very large, the knot appears to result from the “threading” of the N-terminal domain through the above mentioned loop. As for SOTCase, the hydrophobicity profile (see Figure S3) does not provide a definite indication that the loop region takes part to contacts aiding the kinetic accessibility of the knotted native state.

**Figure 4:**
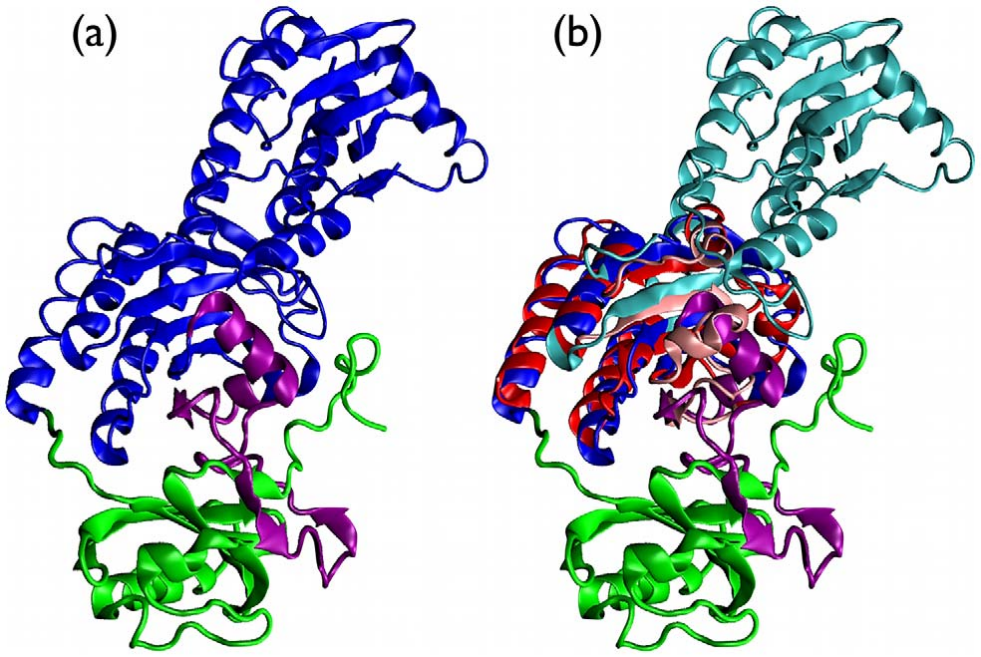
Knotted photosensory core module. PaBphP (a) and its alignment with the unknotted chain 2b18A (b). In the knotted structure the knot-promoting loop is highlighted in purple, while the N-terminal domain, which threads through the loop, is shown in green. In the bottom panel, the aligned residues of knotted and unknotted proteins are colored in blue and red, respectively, while non aligned residues are correspondingly colored in cyan and pink. The N-terminal PAS domain (green) and C-terminal PHY domain (cyan) are well-separated by the aligned region, which instead covers almost completely the central GAF domain of PaBphP photosensory core module.

The removal of the loop, as readily seen from Fig. 4, leads to an unknotted structure, and therefore suggests that, like the other cases, it could be profitably targeted in mutagenesis experiments to ascertain its role in the process of knot formation.

### Other correspondences of knotted and unknotted proteins

The above analysis was based on the identification of knot-promoting regions suggested by significant alignments of the knotted representatives in Table 1 against unknotted representatives. Only for the three representatives discussed above it was possible to identify such correspondences on the basis of available structural data.

Yet, it is interesting to point out that for two other representatives, namely chains 2etlA (ubiquitin carboxy-terminal hydrolase, UCH) and 2p02A (alpha subunit of human S-adenosylmethionine synthetase, hereafter 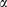-SAM-S), good structural matches involving the knotted region were found against unknotted structures. At variance with previous cases, however, these matches do not suggest the possibility to unknot the protein by a simple excision operation. Yet, they are interesting for the purpose of understanding how continuous is the structure space between knotted and unknotted PDB entries.

**Figure 5:**
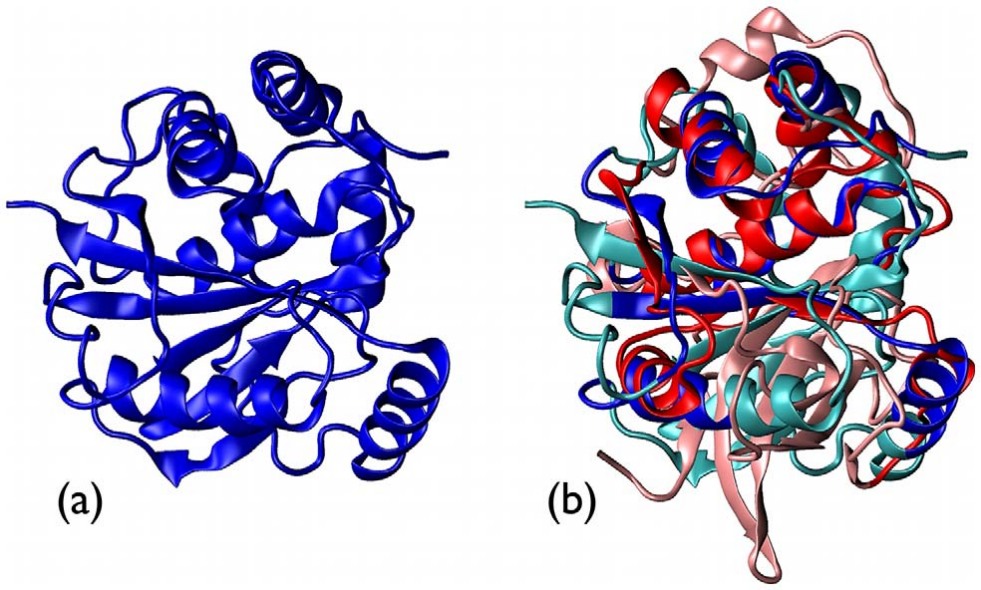
Knotted protein. UCH (a) and its alignment with the unknotted chain 1aecA (b). The aligned residues of the knotted and unknotted protein are colored in blue and red, respectively while unsaturated colors (cyan and pink) are used for non-aligned residues.

The two examples are shown in Fig. 5. Panel (b) presents a superposition of the knotted UCH [45], which is the only 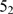 knot representative, against the unknotted entry 1aecA [46]. The alignment, though not spanning the entirety of the protein structures, highlights a good correspondence of secondary and tertiary structure elements.

**Figure 6:**
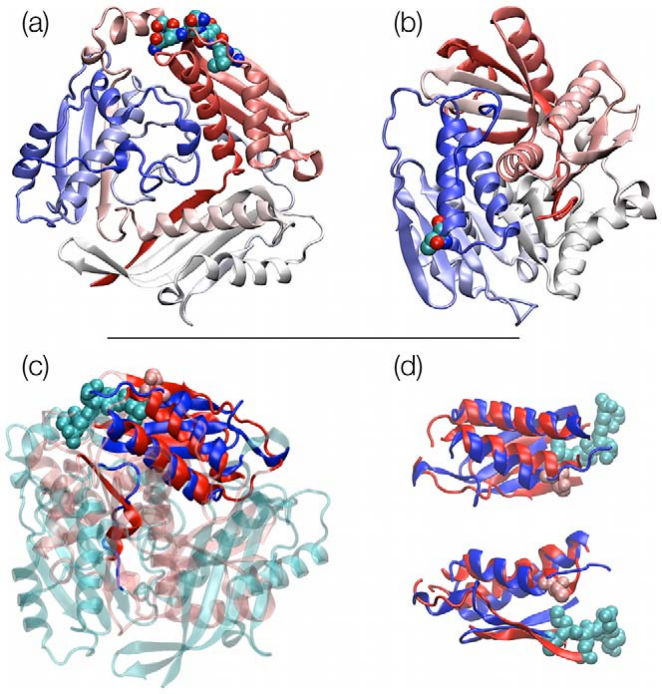
Knotted protein. 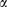
-SAM-S (a) and unknotted protein 1b×4A (b), colored according to the residue index (red-white-blue); bottom, the structural superposition of these two entries where the aligned residues of knotted and unknotted proteins in the bottom row are colored in blue and red, respectively, while non aligned residues are correspondingly colored in cyan and pink. Panel (c) shows the whole structures, while in panel (d) two orthogonal views of the sole aligned regions are presented. In all panels catalytic residues are included in Van der Waals representation. In panel (a), the knotted topology of 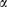-SAM-S can be readily perceived following the coloring of the chain.

Analogous considerations, hold for the alignment of 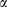-SAM-S [47] and 2b×4A [48] (Fig. 6), whose mutual sequence identity is less than 10%. The alignment highlights the threefold symmetry of the knotted protein, which however, builds on a non-trivial domain organization which results in a trefoil knot.

### Conclusions

In this study we presented a database-wide comparative analysis of pairs of knotted and unknotted proteins. The study was aimed at understanding if, and to what extent, the rare instances of known knotted proteins are discontinuously related in sequence or structure space to unknotted proteins.

The analysis proceeded by first identifying minimally-redundant sets for the 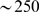 knotted protein chains found among the presently-available PDB entries. Specifically, the latter were found to be fully represented by 11 entries. These non-homologous and structurally-different representatives cover all the 4 different types of knots which have been found to date in proteins. Most of the represented knots are chiral. Excluding from considerations a trefoil-knotted protein whose origin is probably artifactual, it is found that left- and right-handed chiral knots are almost equally represented. This fact, which had not been pointed out before, is well compatible with the null hypothesis that left- and right-handed protein knots occur in equal proportions in non-redundant datasets.

In order to understand what type of primary sequence relatedness exists between knotted and unknotted proteins, a PDB-wide BLAST [35] search was performed for each of the knotted representatives to identify the sequence homologs. For nearly all of the representatives, the analysis did not return significant sequence-based matches with unknotted proteins. One notable exception was constituted by a specific SOTCase, namely 2fg6C, whose phylogenetic tree comprises both knotted and unknotted entries. The knotted homologs fully occupied two commonly-rooted sub-branches of the tree, suggesting the existence of a single evolutionary event at the basis of the divergence of the knotted group from the main unknotted tree. The structural alignment of members of the knotted SOTCase phylogenetic tree highlighted that the knotted domains differed from the unknotted counterparts, for the presence of two additional short segments with a small end-to-end separation. The bridging of these knot-promoting loop segments, one of which was identified in ref. [19] using a different approach, that is their removal from the primary sequence, ought to result in an unknotted native state equivalent to the one of the unknotted homologs.

The insight offered by the sequence comparative investigation was finally complemented by one based on pairwise structural alignments. At variance with the sequence case, the structural one revealed several significant knotted/unknotted correspondences. In an appreciable number of instances, these correspondences involved a substantial fraction of the region where the knot is accommodated. Also in these cases, knotted proteins appeared to differ from the unknotted partner by the presence of knot-promoting segments analogous to those identified in the alignments involving the SOTCase. The results therefore point to the key role that these specific, local, protein segments play for the global knotted topology of the folded protein.

These regions might represent ideal candidates for mutagenesis or excision experiments to monitor the impact of these regions on the process of knot formation.

## Materials and Methods

### PDB dataset processing

The PDB database as of December 2009 contained 6.2 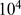 entries, which were parsed into single chains. From the resulting dataset we retained only those chains with length matching the nominal one (provided in the SEQRES PDB field) to within 25 amino acids. Very short (less than 50 a.a.) and very long (more than 1000 a.a.) chains, as well as those with missing 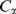 coordinates were not considered. This sieving procedure returned 1.2 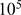 chains.

Detecting and characterizing the presence of knots in proteins requires a suitable generalization of the mathematical notion of knottedness [49]–[51]. The latter is rigorously defined only for circular, closed, chains [52], [53].

In such contexts, at variance with the case of linear open-ended polymers such as proteins, knots cannot be untied by any manipulation preserving the connectivity and self-avoidance of the circular chain. The mathematical concept of knottedness can be extended to protein chains whenever a simple, non-ambiguous way exists to bridge the two termini, such as by prolonging them into an arc that does not intersect the protein hull. Such virtual circularization procedures are actually possible for most protein chains because the N and C termini are usually exposed at the protein surface.

The closure algorithm applied here first performs the identification of those chains with both termini exposed on the surface: this condition is satisfied if one can pass a plane through each terminus, such that all other residues occupy only one of the two subspaces created by the plane. In these cases the chain can be unambiguously closed by adding a segment connecting the termini “at infinity” without intersecting the protein chain.

As many as 6.4 10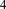 chains could be circularised with this procedure. For proteins constituted by identical monomeric chains, only one representative chain was considered, reducing the number of considered entries to 4.5 10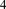.

### Topological classification and culling

The dataset of the 4.5 10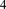 circularised protein chains was further processed to establish the knot topology of each entry; the knot type was determined using the scheme of refs. [12], [13] which is based on the KNOTFIND algorithm.

Only 247 protein chains were found to have nontrivial topology. These two sets are affected by a large sequence redundancy, which was removed at the stringent 10% sequence identity level using the web tool developed by Cedric Notredame and available at http://www.expasy.ch/tools/redundancy. The culling procedure returned the 11 representatives shown in Table 1. No significant structural relatedness was found among any pair of these representatives.

The large set of unknotted proteins was processed with the UniqueProt [54] standalone program to efficiently remove the overall sequence similarity. Its iterative application with default parameters returned 2.4 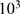 unknotted representatives.

### Structural alignment

The publicly-accessible MISTRAL multiple structural alignment tool [40] was used for the systematic structural comparison of knotted and unknotted proteins. The alignment tool was used for two reasons. First, it has been shown to yield a reliable estimate of the statistical significance of a given alignment and, secondly, it can detect structurally-corresponding regions that do not have the same succession or directionality along the primary sequence of the input proteins. The necessity to account for such generalised relationships in proteins has emerged recently [55]. It appears particularly relevant in this context given the expected difficulty in establishing overall correspondences of knotted and unknotted proteins from a standard (sequential) sequence-based perspective.

All pairwise structural alignments between the representatives of the unknotted and knotted proteins were computed. Among those with a 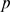-value smaller than 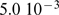 we singled out those which involved at least 40% of the protein region that encompasses the knot. The latter is defined by taking the chain portion that is strictly occupied by the knot according to the criterion of ref. [50] and extending it by 20 amino acids on both sides of the primary sequence (unless a terminus is closer). The selected alignments are provided in Table S2.

## Supporting Information

Figure S1 Hydrophobicity profile of the knotted protein 2fg6C.
(0.24 MB PDF)

Figure S2 Hydrophobicity profiles for the knotted protein 2ha8A.
(0.29 MB PDF)

Figure S3 Hydrophobicity profile for the knotted protein 3c2wH.
(0.18 MB PDF)

Table S1 List of knotted protein chains.
(0.06 MB PDF)

Table S2 Top ranking MISTRAL alignments of knotted and unknotted representatives.
(0.06 MB PDF)
