# Supplementary material for: Knotted vs. Unknotted Proteins: Evidence of Knot-Promoting Loops
Source: PLoS Comput Biol. 2010 Jul 29;6(7):e1000864. doi: 10.1371/journal.pcbi.1000864 (PMC2912335; doi:10.1371/journal.pcbi.1000864)
Supplement: Figure S1 — Hydrophobicity profile of the knotted protein 2fg6C. (0.24 MB PDF) [file pcbi.1000864.s001.pdf]

Supporting Information - figure S1

"Knotted vs. unknotted proteins: evidence of knot-promoting loops"

Raffaello Potestio[1], Cristian Micheletti[1,2,3,*], Henri Orland[4]
1 SISSA - Scuola Internazionale Superiore di Studi Avanzati, via Bonomea 265, 34136 Trieste, Italy
2 DEMOCRITOS CNR-IOM
3 Italian Institute of Technology (SISSA unit)
4 Institut de Physique Théorique, CEA, F-91191 Gif-sur-Yvette, France
∗ *E-mail: michelet@sissa.it*

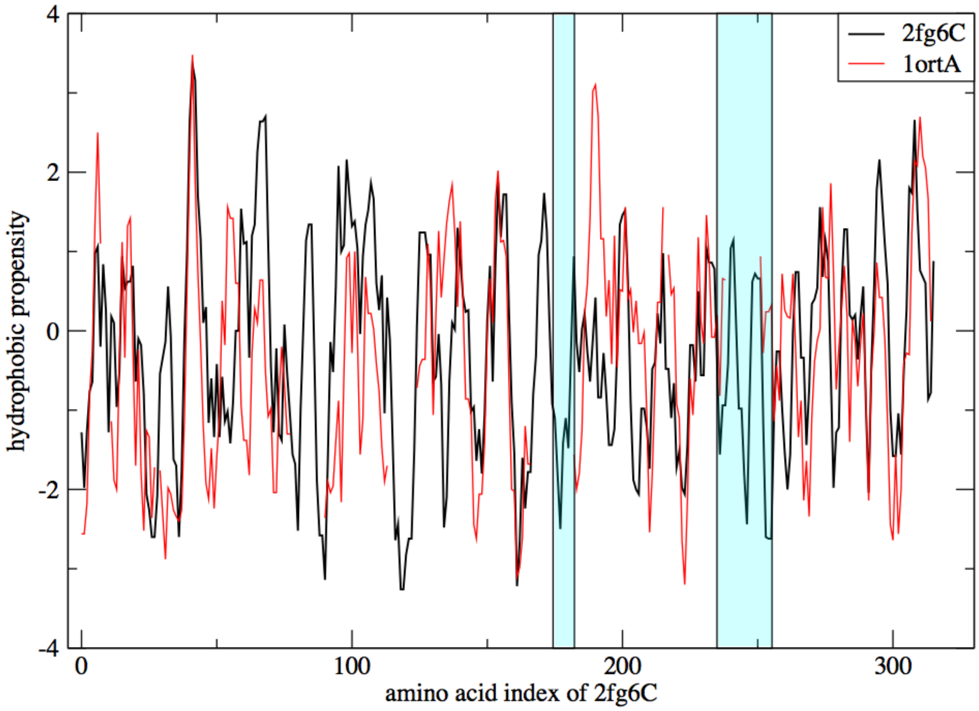

Hydrophobicity profiles of the knotted protein 2fg6C and the structurally-matching amino acids of the unknotted partner 1ortA. The knot-promoting segments (174–182, 235–255) are highlighted by the light blue boxes. The hydrophobicity was calculated using the Kyte and Doolittle scale and an averaging window of 5 amino acids.
