# Supplementary material for: Knotted vs. Unknotted Proteins: Evidence of Knot-Promoting Loops
Source: PLoS Comput Biol. 2010 Jul 29;6(7):e1000864. doi: 10.1371/journal.pcbi.1000864 (PMC2912335; doi:10.1371/journal.pcbi.1000864)
Supplement: Figure S2 — Hydrophobicity profiles for the knotted protein 2ha8A. (0.29 MB PDF) [file pcbi.1000864.s002.pdf]

Supporting Information - figure S2

"Knotted vs. unknotted proteins: evidence of knot-promoting loops"

Raffaello Potestio[1], Cristian Micheletti[1,2,3,*], Henri Orland[4]
1 SISSA - Scuola Internazionale Superiore di Studi Avanzati, via Bonomea 265, 34136 Trieste, Italy
2 DEMOCRITOS CNR-IOM
3 Italian Institute of Technology (SISSA unit)
4 Institut de Physique Théorique, CEA, F-91191 Gif-sur-Yvette, France
∗ E-mail: michelet@sissa.it

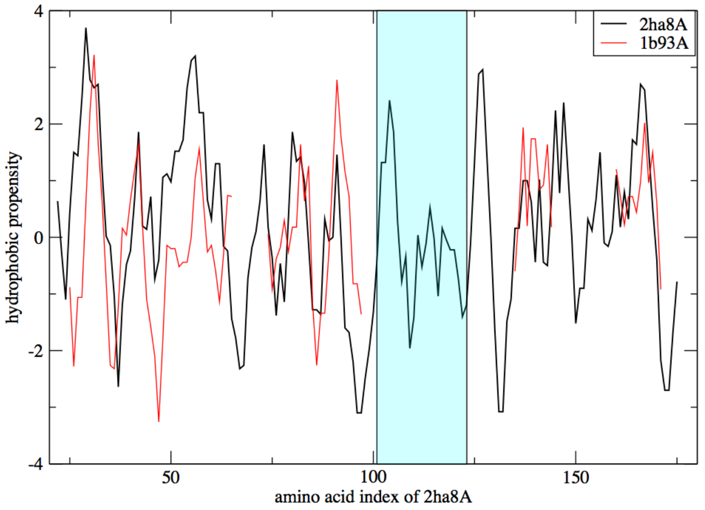

a)

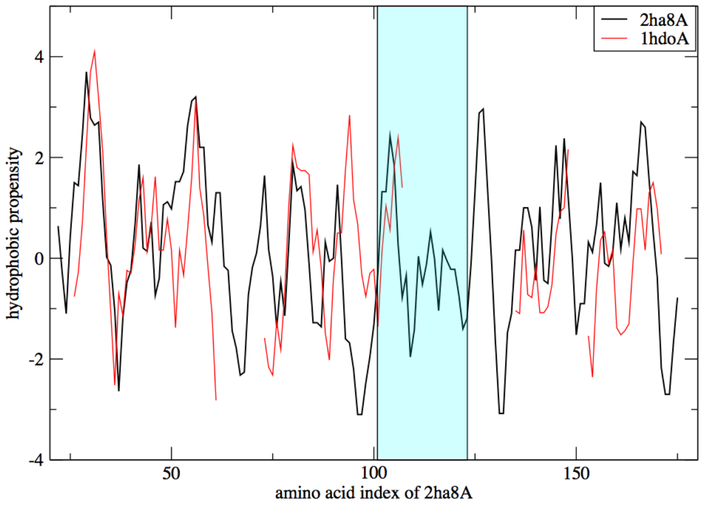

b)

Hydrophobicity profiles for the knotted protein 2ha8A and the MISTRAL structurally-matching amino acids of the unknotted protein 1b93A (panel a) and 1hdoA (panel b). The knot-promoting segment (101–123) is highlighted by the light blue box. The hydrophobicity was calculated using the Kyte and Doolittle scale and an averaging window of 5 amino acids. Notice that, at variance with the case in panel a, the MISTRAL alignment of 2ha8A and 1hdoA, panel b, is non-sequential (see diagram in Fig. 3 of our paper) and shows two gaps, one of which is the knot-promoting segment.
