# Supplementary material for: Knotted vs. Unknotted Proteins: Evidence of Knot-Promoting Loops
Source: PLoS Comput Biol. 2010 Jul 29;6(7):e1000864. doi: 10.1371/journal.pcbi.1000864 (PMC2912335; doi:10.1371/journal.pcbi.1000864)
Supplement: Figure S3 — Hydrophobicity profile for the knotted protein 3c2wH. (0.18 MB PDF) [file pcbi.1000864.s003.pdf]

Supporting Information - figure S3:

"Knotted vs. unknotted proteins: evidence of knot-promoting loops"

Raffaello Potestio[1], Cristian Micheletti[1,2,3,*], Henri Orland[4]

1 SISSA - Scuola Internazionale Superiore di Studi Avanzati, via Bonomea 265, 34136 Trieste, Italy

2 DEMOCRITOS CNR-IOM

3 Italian Institute of Technology (SISSA unit)

4 Institut de Physique Théorique, CEA, F-91191 Gif-sur-Yvette, France

∗ *E-mail: michelet@sissa.it*

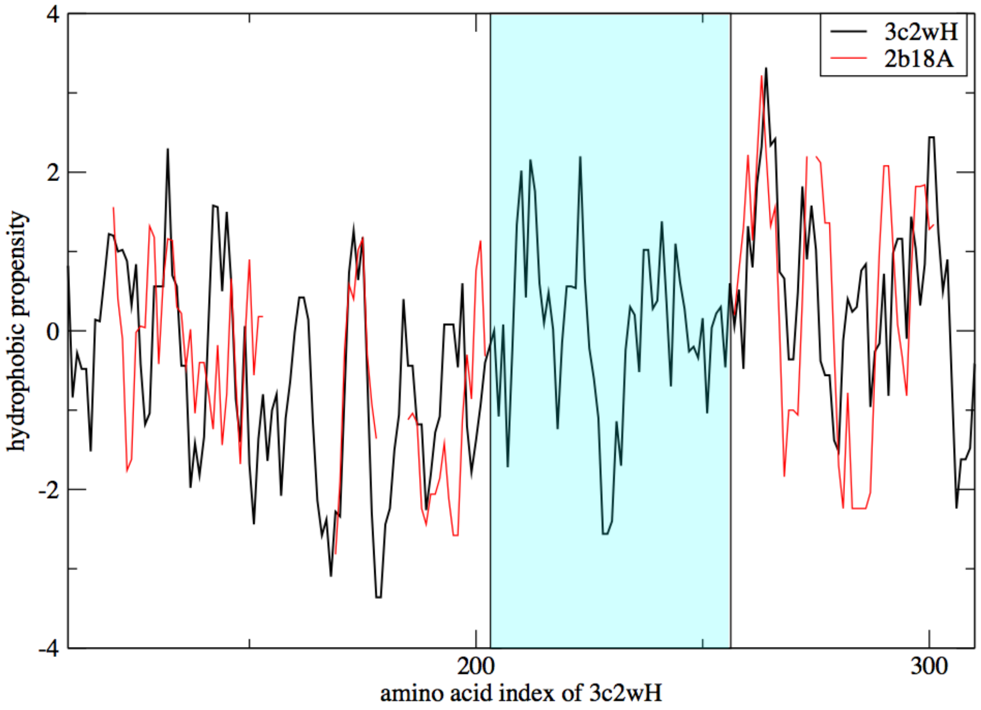

Hydrophobicity profiles for the knotted protein 3c2wH and the MISTRAL structurally-matching amino acids of the unknotted chain 2b18A. The knot-promoting segment (203–256) is highlighted by the light blue box.
