# Supplementary material for: Knotted vs. Unknotted Proteins: Evidence of Knot-Promoting Loops
Source: PLoS Comput Biol. 2010 Jul 29;6(7):e1000864. doi: 10.1371/journal.pcbi.1000864 (PMC2912335; doi:10.1371/journal.pcbi.1000864)
Supplement: Table S1 — List of knotted protein chains. (0.06 MB PDF) [file pcbi.1000864.s004.pdf]

Supporting Information - table S1

“Knotted vs. unknotted proteins: evidence of knot-promoting loops”

Raffaello Potestio[1], Cristian Micheletti[1,2,3,*], Henri Orland[4]
1 SISSA - Scuola Internazionale Superiore di Studi Avanzati, via Bonomea 265, 34136 Trieste, Italy
2 DEMOCRITOS CNR-IOM
3 Italian Institute of Technology (SISSA unit)
4 Institut de Physique Théorique, CEA, F-91191 Gif-sur-Yvette, France
∗ *E-mail: michelet@sissa.it*

1a42A	1am6A	1azmA	1bcdA	1bicA	1bnqA	1bnuA	1bnwA	1bv3A	1bzmA
1cahA	1caiA	1cajA	1cakA	1calA	1camA	1cayA	1cazA	1cilA	1cngA
1craA	1czmA	1dmxA	1dmyA	1eouA	1fljA	1fqnA	1fqrA	1fr4A	1fsqA
1fsrA	1g0eA	1g0fA	1g1dA	1g3zA	1g45A	1g46A	1g48A	1g4jA	1g4oA
1g52A	1g54A	1gz0A	1gz0B	1gz0D	1gz0F	1gz0H	1hcbA	1heaA	1hecA
1huhA	1i8zA	1i90A	1i91A	1i9lA	1i9mA	1i9nA	1i9oA	1i9pA	1i9qA
1if4A	1if5A	1if6A	1if7A	1if8A	1if9A	1ipaA	1j9wA	1jv0A	1keqA
1kwqA	1kwrA	1lg5A	1nxzA	1nxzB	1o6dA	1oq5A	1p7lA	1qmgA	1qmgD
1rayA	1razA	1rg9A	1rj5A	1rj6A	1rzaA	1rzcA	1rzdA	1rzeA	1s1hI
1t9nA	1tb0X	1tbtX	1te3X	1teuX	1tg3A	1tg9A	1th9A	1thkA	1ttmA
1ugaA	1ugcA	1ugdA	1ugeA	1ugfA	1uggA	1urtA	1v9eA	1v9iC	1vh0A
1x7pA	1xd3A	1xd3C	1xegA	1xevA	1xevB	1xpzA	1xq0A	1yddA	1yh1A
1yo0A	1yo1A	1yo2A	1yveI	1z97A	1zgeA	1zgfA	1zh9A	1zjrA	1zsaA
1zsbA	2aw1A	2ax2A	2cbaA	2cbbA	2cbcA	2cbdA	2cbeA	2efvA	2egvA
2etlA	2eu2A	2eu3A	2ez7A	2fg6C	2fg6D	2fg6Z	2fg7C	2fg7X	2fnkA
2fnmA	2fnnA	2foqA	2fosA	2fovA	2foyB	2g7mC	2g7mX	2gehA	2h15A
2ha8A	2hd6A	2hfxA	2hfyA	2hkkA	2hl4A	2hocA	2nmxA	2nmxB	2nn1A
2nn1B	2nn7A	2nngA	2nnoA	2nnsA	2nnvA	2nwoA	2nwpA	2nwyA	2nwzA
2nxrA	2nxsA	2nxtA	2o9cA	2obvA	2osfA	2osmA	2p02A	2pouA	2povA
2qmmA	2qo8A	2qp6A	2rh3A	2vvbX	2wegA	2wehA	2wejA	3b4fA	3bbdA
3bbeA	3bbhA	3betA	3bjxB	3bl0A	3c2wC	3c2wH	3c7pA	3cajA	3czvB
3d0nA	3d93A	3d9zA	3da2A	3dbuA	3dc9A	3dccA	3dcsA	3dd0A	3dd8A
3dv7A	3dvbA	3dvcA	3dvdA	3eftA	3f4xA	3f8eA	3ffpX	3gz0A	3hkqA
3hkuA	3hs4A	3iaiA	3iaiB	3iaiD	3ibiA	3iblA	3ibnA	3ibuA	3ic6A
3iefA	3ilkA	3k2fA	3ktyB	3ktyC	4cacA	5cacA			

List of knotted protein chains. The 247 listed entries were extracted from the December 2009 PDB release, using the procedure described in the Materials and Methods section of the main article.
