# Supplementary material for: Knotted vs. Unknotted Proteins: Evidence of Knot-Promoting Loops
Source: PLoS Comput Biol. 2010 Jul 29;6(7):e1000864. doi: 10.1371/journal.pcbi.1000864 (PMC2912335; doi:10.1371/journal.pcbi.1000864)
Supplement: Table S2 — Top ranking MISTRAL alignments of knotted and unknotted representatives. (0.06 MB PDF) [file pcbi.1000864.s005.pdf]

Supporting Information - table S2

"Knotted vs. unknotted proteins: evidence of knot-promoting loops"

Raffaello Potestio[1], Cristian Micheletti[1,2,3,*], Henri Orland[4]

1 SISSA - Scuola Internazionale Superiore di Studi Avanzati, via Bonomea 265, 34136 Trieste, Italy

2 DEMOCRITOS CNR-IOM

3 Italian Institute of Technology (SISSA unit)

4 Institut de Physique Théorique, CEA, F-91191 Gif-sur-Yvette, France

∗ *E-mail: michelet@sissa.it*

knotted protein PDBid	unknotted protein PDBid	fraction of aligned knot residues
2fg6C	1ortA	0.706
2ha8A	2qipA	0.647
2ha8A	1b93A	0.565
2ha8A	1f51E	0.494
1vh0A	1b93A	0.596
2efvA	2rjiA	0.531
2ha8A	3fhkA	0.412
3bbeA	1aoxA	0.443
2ha8A	1u7oA	0.459
2ha8A	2b98A	0.494
2fg6C	1a4iA	0.560
1vh0A	1d0iA	0.426
1vh0A	2z5vA	0.468
2qmmA	1hdoA	0.651
2ha8A	1hdoA	0.553
2ha8A	1d0iA	0.518
2ha8A	3gpgA	0.518
2efvA	1d8jA	0.494
2ha8A	1c25A	0.647
3ktyC	121pA	0.413

Top ranking MISTRAL alignments of knotted and unknotted representatives. In order to account for the different topology of the compared proteins, the alignments were obtained with the following non-default MISTRAL parameters: the alignment tolerance was set to 6.0 Å; the minimum segment length was set to 10 amino acids. For each alignment we report, in the third column, the percentage of the knotted region (defined in Table I of the main article) that takes part to the structural alignment. The listed pairs include only MISTRAL alignments with p-value $\leq 5\,10^{-3}$, where the percentage of the aligned knotted region is larger than 40.
